# Impact of bariatric surgery on obstructive sleep apnea severity and continuous positive airway pressure therapy compliance—prospective observational study

**DOI:** 10.1038/s41598-021-84570-6

**Published:** 2021-03-02

**Authors:** Paweł Nastałek, Kamil Polok, Natalia Celejewska-Wójcik, Aleksander Kania, Krzysztof Sładek, Piotr Małczak, Piotr Major

**Affiliations:** 1 Department of Pulmonology, 2nd Department of Internal Medicine, Jagiellonian University Medical College, ul. Jakubowskiego 2, 30-688 Kraków, Poland; 2 2nd Department of General Surgery, Jagiellonian University Medical College, ul. Jakubowskiego 2, 30-688 Kraków, Poland

**Keywords:** Respiratory tract diseases, Outcomes research, Obesity

## Abstract

To evaluate association between bariatric surgery and changes in obstructive sleep apnea (OSA) severity and sleep architecture was as well as to asses continuous positive airway pressure (CPAP) effectiveness and compliance. We enrolled patients undergoing bariatric surgery. Polysomnography was performed in each patient preoperatively and 12 months after the procedure in a subgroup of patients diagnosed with OSA. STOP-BANG, Epworth Sleepiness Scale (ESS) and Berlin questionnaire scores were obtained pre- and postoperatively. CPAP compliance data was recorded during follow-up hospitalization. Among 44 patients with median age of 49.5 years, predominantly women (68.2%) pre- and postoperative polysomnography was performed. We observed significant improvement in STOP-BANG (6.0 vs. 3.0, p < 0.001) and ESS (12.0 vs. 5.0, p < 0.001) scores, apnea–hypopnea index (44.9 vs. 29.2, p < 0.001), oxygen desaturation index (43.6 vs. 18.3, p < 0.001) and sleep architecture parameters. CPAP compliance was poor with a median percentage of days with CPAP use accounting to 49.3%. Bariatric surgery is associated with a significant decrease in the number of sleep-related respiratory disturbances, as well as improvement of sleep efficiency. Postoperative CPAP therapy compliance was poor despite low rate of OSA resolution. This study suggests that patients with OSA undergoing bariatric surgery require postoperative reassessment.

## Introduction

Obesity represents a major global health challenge with estimated 650 million cases worldwide^1^. Its prevalence varies significantly depending on a country and ranges from 3.7% in Japan to 38.2% in the United States^2,3^. Obesity is a key risk factor for multiple diseases such as hypertension, diabetes, stroke, osteoarthritis and cancer, thus leading to premature disability and death^4^. It is considered the most important, best documented and one of the few modifiable risk factors for obstructive sleep apnea (OSA).

OSA is the most common manifestation of sleep breathing disorders and affects 4% males and 2% females in the general population^5,6^. Untreated OSA is associated with a decline in quality of life, development of several cardiovascular comorbidities, increased risk of car accidents and premature mortality^7^. The gold standard for OSA treatment is continuous positive airway pressure (CPAP)^5^. Meta-analyses of randomized controlled trials proved the effectiveness of CPAP in attenuation of sleep apnea, reduction of OSA symptoms and OSA-associated comorbidity^8^.

Bariatric surgery is currently considered the most effective modality of obesity treatment, associated with long-term maintenance of body weight reduction^9^. It is indicated in patients with BMI > 40 kg/m^2^ or BMI > 35 kg/m^2^ accompanied by comorbidities i.e. heart failure, type 2 diabetes, hypertension and OSA^10^. Untreated OSA in obese individuals qualified for bariatric surgery is related to increased risk of complications in perioperative period^11^. Preoperatively initiated and effective CPAP therapy reduces the risk of complications associated with general anesthesia as well as the bariatric procedure itself^12^.

Despite the beneficial effects of weight reduction on sleep breathing disorders confirmed by number of studies, there is still significant uncertainty whether bariatric surgery eliminates OSA and allows CPAP therapy discontinuation. This study was an attempt to elucidate above mentioned issues. The primary aim of this study was to assess the association between bariatric surgery and the severity of sleep-related disturbances.

## Materials and methods

### Study design

This was a single-centre observational study including obese patients undergoing primary bariatric surgery. Preoperative evaluation of OSA risk using validated screening tools and polysomnography were routinely performed in the Pulmonology Department. If a patient met obstructive sleep apnea diagnostic criteria and required PAP therapy, CPAP was introduced and titrated. After the index procedure in the 2nd Department of Surgery each patient diagnosed with OSA was referred to the Pulmonology Department for follow-up reassessment within 12 months after the index procedure including sleep questionnaires and polysomnography. Moreover, the CPAP compliance data from patients` CPAP devices was downloaded and analysed. If persistent sleep apnea requiring CPAP therapy was present in follow-up polysomnography, we performed a re-titration of therapeutic CPAP.

Patients that finally did not undergo surgery, who did not meet the criteria of OSA (apnea–hypopnea index [AHI] < 5/h) or missed the follow up hospitalisation were excluded from final analysis. The data was collected between December 2014 and May 2018. The study complies with the Declaration of Helsinki. All patients gave informed consent to participate in the study and official approval for the study protocol from the Jagiellonian University Ethics Committee was obtained (KBET/126/B/2014).

### Study subjects’ characteristics

On admission to the Pulmonology Department clinical characteristics of study subjects including demographic data (age, gender), physical examination (blood pressure, heart rate, haemoglobin saturation, patient`s height and weight) and comorbidities (history of diabetes, pre-diabetes, hypertension, atrial fibrillation, chronic heart failure, coronary artery disease, osteoarthritis, smoking) was obtained. American Society of Anaesthesiologists Classification (ASA) score records were gathered from 2nd Department of General Surgery for individuals who eventually underwent bariatric surgery procedure.

### Questionnaires

Patients were assessed using validated screening tools: STOP-BANG questionnaire, Berlin questionnaire and Epworth Sleepiness Scale (ESS)^13–15^. The STOP-BANG questionnaire includes four questions related to snoring (S), tiredness (T), observed apnea (O), high blood pressure (P) and additional anatomical and demographic queries; BMI (B), age (A), neck circumference (N) and male gender (G). For each question, answering “yes” scores 1, a “no” response scores 0, with cut-off score for increased OSA risk ≥ 3^13^. The Berlin questionnaire is a self-administered questionnaire which consists of 10 questions in three categories related to the presence and severity of snoring, frequency of daytime sleepiness, and the presence of obesity or hypertension. In category one, high risk was defined as persistent symptoms in two or more questions related to snoring. In category two, high risk was defined as persistent daytime sleepiness, drowsiness while driving, or both. In category three, high risk was defined as a history of hypertension or a BMI higher than 30 kg/m^2^. High-risk subjects for OSA were those who were defined as high risk in at least two out of three categories^14^. ESS is a simple, self-administered questionnaire which is shown to provide a measurement of the subject's general level of daytime sleepiness during certain activities (watching TV, reading a book, travelling, driving etc.). Patient evaluated chance of dozing scoring from ‘’0′’ that indicates “no chance of dozing” to “3” that means high dozing chance. Result ≥ 10 indicates excessive daytime sleepiness that might be associated with OSA^15^.

### Obstructive sleep apnea diagnosis and treatment

Polysomnography was performed using a 31-channel polysomnographic system (Phillips ALICE 6LDe) system. Detection of apneas and hypopneas as well as diagnosis of OSA and its severity assessment were performed in accordance with current American Academy of Sleep Medicine criteria^16^. Each polysomnography was assessed by a pulmonologist certified in sleep medicine. Titration of therapeutic CPAP was performed using automatic CPAP machine (Philips Respironix REMstar Auto A-flex Series 60 device) coupled with polysomnography. CPAP recordings were analysed using Philips Encore Software.

### Bariatric surgery

The surgical technique has been described in detail previously^17^. A Veress needle was used to achieve pneumoperitoneum (15 mmHg). The routine procedure required insertion of four trocars during laparoscopic sleeve gastrectomy (LSG) and five trocars during laparoscopic Roux-en-Y gastric bypass (LRYGB). A sealer/divider or ultrasonic shears were used for dissection and coagulation. A 34-French gastric bougie was used to calibrate the gastric sleeve. Gastrectomy started 4–5 cm proximal to the pylorus with continuously applied linear staples straight to the angle of His. The staple line was reinforced by a running suture. The resected portion of the stomach was removed from the peritoneal cavity through the left flank trocar site during LSG. LRYGB required creation of a pouch by one horizontal 45-mm stapler followed by vertical stapling toward the angle of His, until the pouch was totally separated from the rest of the stomach. A gastrojejunal anastomosis was created with a linear stapler with hand-sewn closure of the remaining defect. The length of the alimentary and enzymatic limb was standardized in all patients as 150 and 100 cm, respectively. A jejunojejunal anastomosis was created using a linear stapler. A routine 10- or 12-mm port site closure was performed to prevent herniation. Perioperative care in our department is based on ERAS guidelines^18,19^.

### Statistical analysis

Categorical variables were presented as counts (%) and compared using chi-square test, while continuous variables were presented as median (interquartile range) and compared using Mann–Whitney U test unless otherwise specified. Differences between dependent continuous variables with non-normal distribution were assessed using Wilcoxon test. Correlation between two variables with non-normal distribution was assessed using Spearman`s Rank test. A two-sided p-value of 0.05 was considered statistically significant. It was a complete-case analysis. Patients with missing postoperative BMI values (n = 3) were excluded from analyses including this variable. Statistical analysis was performed using Statistica 13 software. Graphs were created with GraphPad Prism 8.3.

### Ethics approval

Approval for the study protocol was obtained from the Jagiellonian University Ethics Committee was obtained (KBET/126/B/2014).

### Consent to participate

All patients signed an informed consent.

## Results

### Study group characteristics

**Figure 1:**
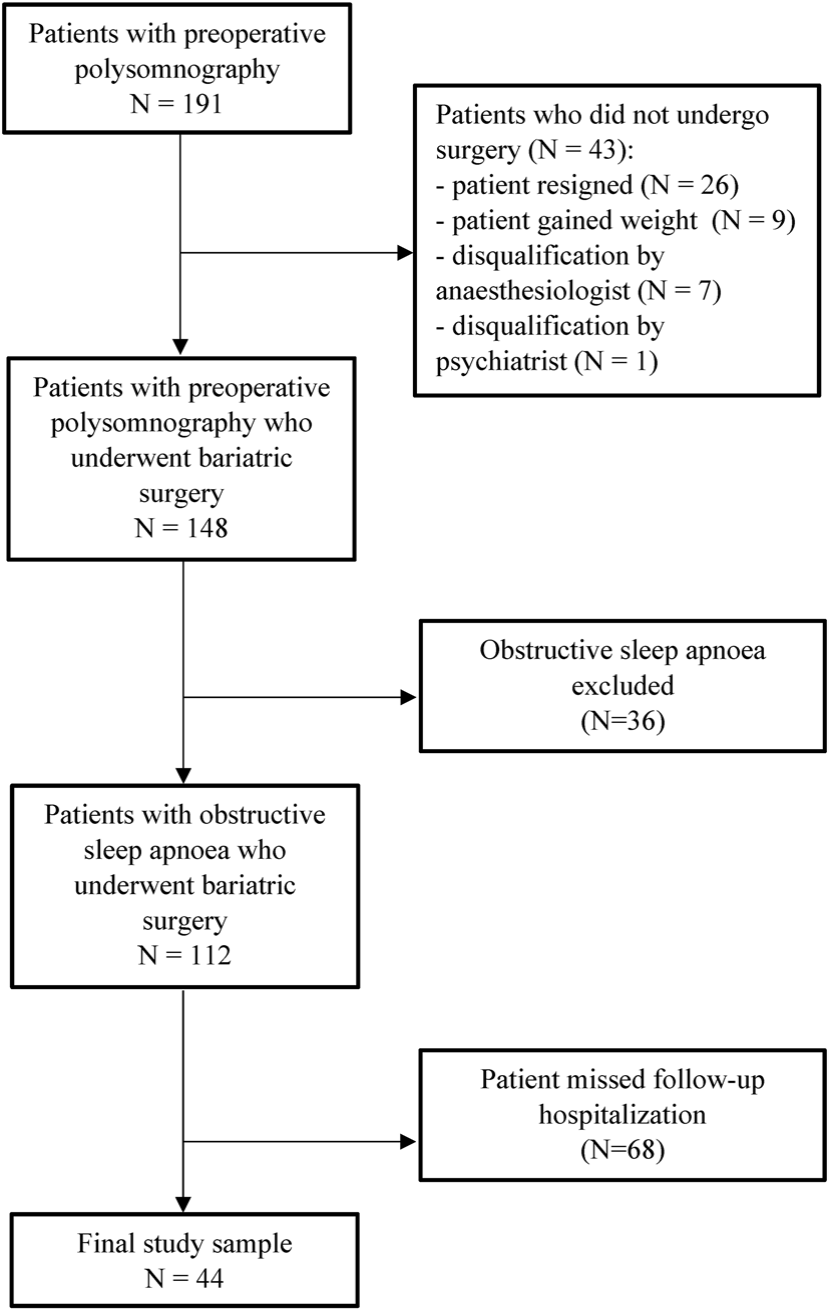
Study flowchart. N, number of patients.

We enrolled 191 patients with preoperative polysomnography, of whom 43 (22.5%) patients eventually did not undergo bariatric surgery. Among the remaining 148 individuals who underwent bariatric surgery, 112 (75.7%) patients were diagnosed with obstructive sleep apnea and were referred to the pulmonology clinic for the follow-up hospitalization, of whom 44 patients (39.3%) were finally reassessed and included in the final analysis. Detailed information can be found in the study flowchart (Fig. 1).

The study group included patients at the median age of 49.5 (41.5–57.5) years, 14 (31.8%) of whom were male. The most common comorbidities were hypertension (31/44, 70.5%), diabetes (18/44, 40.9%) and dyslipidemia (17/44, 38.6%). Majority of patients underwent laparoscopic sleeve gastrectomy (31/44, 70.5%), while laparoscopic Roux-en-Y gastric bypass was performed in the remaining 13 patients (29.5%). Detailed study participant characteristics are presented in Table 1.

**Table 1:** Demographic and clinical characteristics of the study group.

Characteristic	Patients (N = 44)
**Demographic**	
Age (years)	49.5 (41.5–57.5)
Gender, male	14 (31.8%)
**Preoperative assessment**	
Maximal recorded body mass index (kg/m^2^)	47.2 (42.6–53.1)
Body mass index on admission (kg/m^2^)	45.0 (40.8–50.6)
Systolic blood pressure (mmHg)	130.0 (120.0–150.0)
Diastolic blood pressure (mmHg)	80.0 (80.0–90.0)
Heart rate (beats/min.)	78.0 (70.0–88.0)
Hemoglobin oxygen saturation (%)	98.0 (96.0–98.0)
ASA II/ASA III	35 (79.5%)/9 (20.5%)
**Comorbidities**	
Hypertension	31 (70.5%)
Diabetes	18 (40.9%)
Pre-diabetes	2 (4.5%)
Dyslipidemia	17 (38.6%)
Osteoarthritis	16 (36.4%)
Atrial fibrillation	3 (6.8%)
Chronic heart failure	1 (2.3%)
Varicose veins	9 (20.5%)
Smoking	7 (15.9%)
Coronary artery disease	0 (0.0%)
**Scale scores**	
Berlin questionnaire, high risk	41 (93.2%)
STOP-BANG (pts)	6.0 (5.0–7.0)
Epworth Sleepiness Scale (pts)	12.0 (9.0–14.0)
**Hospitalization and surgery data**	
*Type of surgery*	
LSG	31 (70.5%)
LRYGB	13 (29.5%)
Surgery duration (min.)	95.0 (72.5–120.0)
Length of hospitalization (days)	3.0 (2.0–4.0)

ASA, American Society of Anesthesiologists; LSG, laparoscopic sleeve gastrectomy; LRYGB, laparoscopic Roux-en-Y gastric bypass.

### Preoperative sleep Apnea diagnosis and CPAP therapy initiation

Preoperative polysomnography revealed mild, moderate and severe obstructive sleep apnea in 1 (2.3%), 7 (15.9%) and 36 (81.8%) patients respectively.

We observed a significant improvement in AHI (44.9 vs. 3.2, p < 0.001), oxygen desaturation index (ODI; 43.6 vs. 6.7, p < 0.001) and mean SpO_2_ (93.0 vs. 94.0, p < 0.001) during polysomnography coupled with automatic CPAP compared to baseline polysomnography. Normalization of breathing disturbances (defined as AHI < 5/hour) was achieved in 25 (56.8%) patients. The median titrated therapeutic CPAP was 8.3 mbar (7.0–10.0).

### Comparison of pre- and postoperative polysomnography results and scales scores

**Figure 2:**
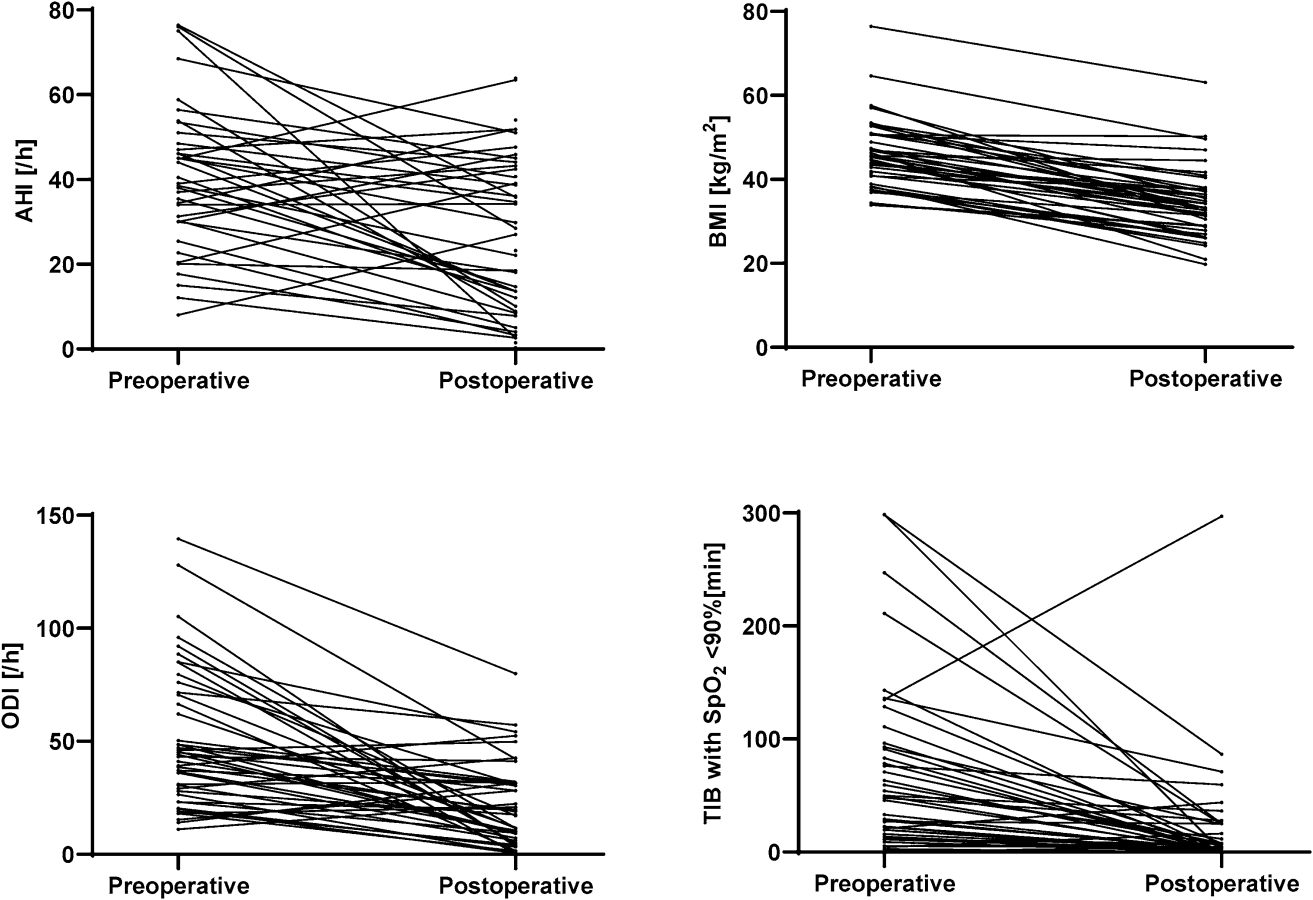
Perioperative changes in apnea–hypopnea index, oxygen desaturation index, body mass index and time in bed with hemoglobin saturation below 90%. AHI, apnea–hypopnea index; BMI, body mass index; ODI, oxygen desaturation index; TIB, time in bed; SpO_2_, hemoglobin oxygen saturation.

**Figure 3:**
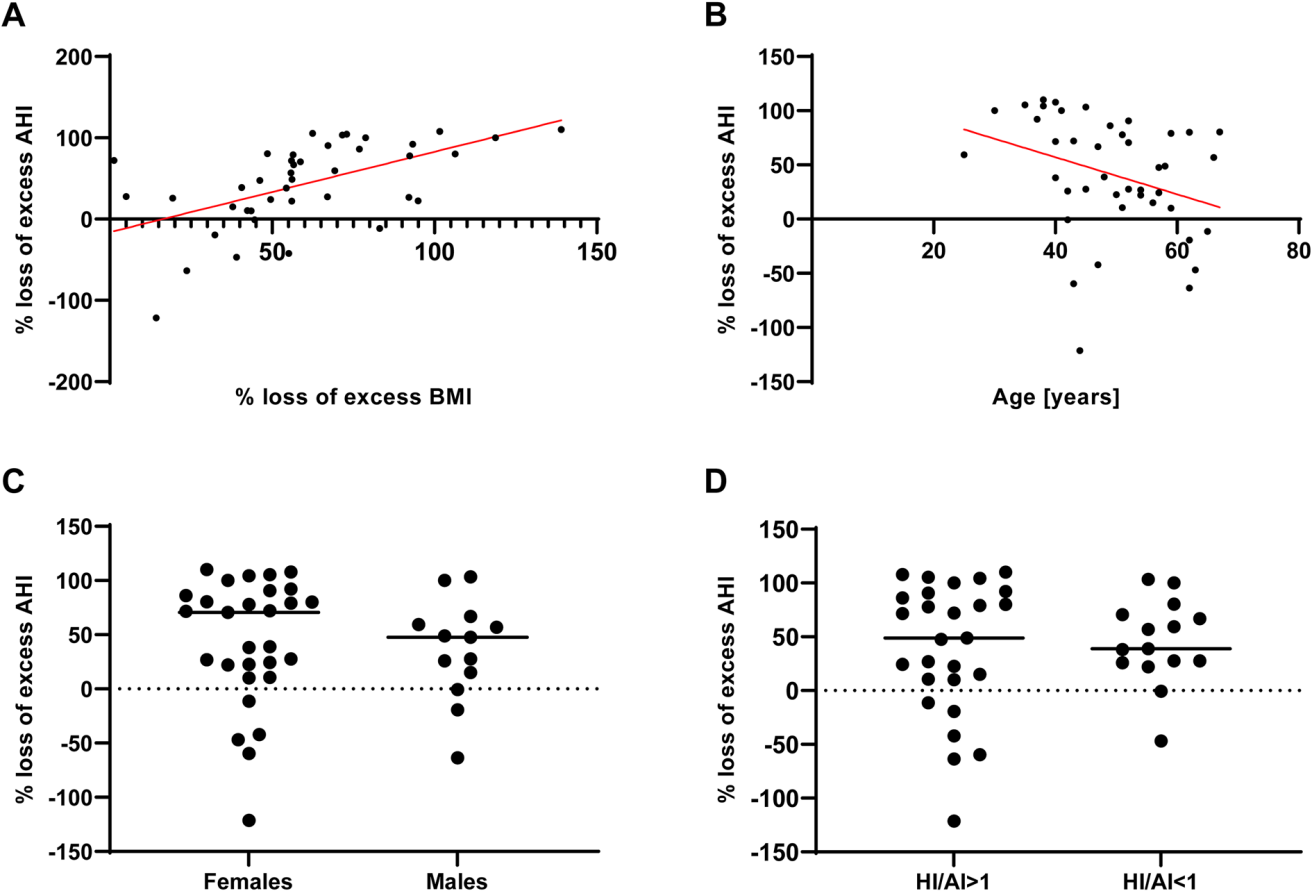
Percentage loss of excess AHI correlated with percentage loss of BMI (**A**), age (**B**) and compared between females and males (**C**) as well as patients with hypopnea index/apnea index ratio > 1 and < 1 (**D**). AI, apnea index; AHI, apnea–hypopnea index; BMI, body mass index; HI, hypopnea index. Red lines in A and B represent lines of best fit, while black horizontal lines in (**C** and **D**) represent median.

We observed a significant improvement in majority of polysomnography parameters, e.g., AHI (44.9 vs. 29.2, p < 0.001), ODI (43.6 vs. 18.3, p < 0.001), mean hemoglobin oxygen saturation (93.0 vs. 95.0, p < 0.001) and snoring (21.6 vs. 4.5%, p < 0.001). Detailed results are summarized in Table 2. Changes in AHI, ODI, BMI and time in bed with haemoglobin saturation below 90% are visualized in Fig. 2. We found no correlation (ρ 0.25, p = 0.11) between absolute change in BMI (postoperative BMI—preoperative BMI) and absolute change in AHI (postoperative AHI—preoperative AHI). The percentage loss of excess AHI was moderately correlated with percentage loss of excess BMI (ρ 0.63, p < 0.001) and weakly negatively correlated with age (ρ − 0.34, p = 0.024). There were no differences in percentage loss of excess AHI between males and females (37.6 vs. 54.7/h, p = 0.39) as well as between patients with the predominance of hypopneas defined as HI/AI index > 1, and those with predominance of apneas, defined as HI/AI index < 1 (49.0 vs. 38.8/h, 0.90). Above mentioned correlations and comparisons are presented in Fig. 3.

**Table 2:** Preoperative and postoperative PSG parameters.

Parameter	Preoperative values	Postoperative values	P value
**Respiratory disturbances**			
AHI (/h)	44.9 (30.8–63.7)	29.15 (11.1–3.65)	< 0.001
OAI (/h)	12.2 (5.1–29.4)	4 (0.5–15.2)	< 0.001
CAI (/h)	1.1 (0.2–2.95)	0.65 (0–2.15)	0.09
AI (/h)	16.5 (5.6–35.3)	6.4 (1.2–17.3)	< 0.001
HI (/h)	24.9 (14.5–29.75)	13.65 (6.9–24.95)	0.007
AHI _REM sleep_ (/h)	44.1 (26.25–70.1)	21.1 (6–52.8)	0.002
AHI _NREM sleep_ (/h)	42.15 (31.45–62.95)	27.55 (9.55–43.9)	< 0.001
AI _REM sleep_ (/h)	18 (6.4–34.15)	5.3 (0–22)	< 0.001
AI _NREM sleep_ (/h)	17.6 (6.7–35.05)	5.2 (1.05–16.45)	0.002
HI_REM sleep_ (/h)	19.55 (11.9–39.6)	11.3 (2.5–24.7)	0.07
HI _NREM sleep_ (/h)	23 (12.5–29.4)	15.5 (6.6–25.7)	0.11
AHI _supine position_ (/h)	49.15 (36.1–71)	29.65 (9.3–47.9)	< 0.001
**Nocturnal pulseoxymetry**			
ODI (/h)	43.6 (27.15–71)	18. 25 (6.65–31.6)	< 0.001
ODI_REM sleep_ (/h)	41.65 (31.55–78.05)	15.8 (4–50.3)	< 0.001
ODI _NREM sleep_ (/h)	41.1 (27.45–65.2)	18 (4.85–33.05)	< 0.001
Mean SpO_2_ (%)	93 (91–94)	95 (93–96)	< 0.001
Minimal SpO_2_ (%)	76 (67.5–82)	83.5 (81–90)	< 0.001
Time with SpO_2_ < 90% (min.)	46.85 (14.75–91.9)	2.75 (0.05–20.4)	< 0.001
Mean HR (beats/min.)	69 (61.75–75.5)	61.1 (56.9–65.3)	< 0.001
Minimal HR (beats/min.)	50 (42–56)	51 (47.1–56)	0.17
Maximal HR (beats/min.)	127.5 (109–154)	109.5 (102–121.3)	0.054
**Sleep architecture**			
Sleep efficiency (%)	73.35 (64.05–87.4)	86.65 (70.7–94.95)	0.016
Sleep latency (min.)	24 (11.9–35.1)	17.25 (10–24.25)	0.11
REM sleep latency (min.)	75.75 (61–114.75)	75 (55–110)	0.65
NREM 1 sleep (%)	22.5 (15.25–29)	26.55 (21.4–33.25)	0.015
NREM 2 sleep (%)	44.25 (35.9–50.45)	45.95 (38.3–53.05)	0.55
NREM 3 sleep (%)	19.15 (12.35–23.35)	13 (7.5–20)	0.003
REM sleep (%)	10 (5.55–14.6)	11.15 (7.45–15.05)	0.50
Respiratory arousals (/h)	16.8 (9.1–35.2)	6.7 (3.2–12.6)	< 0.001
Spontaneous arousals (/h)	9.7 (5.95–14.5)	12 (6.3–22.2)	0.37
Arousals index (/h)	36.85 (17.55–54.3)	23.8 (13.45–45.45)	0.030
Snoring (% of sleep time)	21.6 (15–31.1)	4.45 (0.15–12.15)	< 0.001

AI, apnea index; AHI, apnea–hypopnea index; CAI, central apnea index; HI, hypopnea index; HR, heart rate; NREM, non-rapid eye movement sleep; OAI, obstructive apnea index; ODI, oxygen desaturation index; REM, rapid eye movement sleep ;SpO_2_, hemoglobin oxygen saturation.

Bariatric surgery was associated with significant improvement of sleep efficiency (73.4% vs 86.7%, p = 0.016) and significant decrease in number of respiratory related arousals (16.8 vs 6.7, p < 0.001) and total number of arousals (36.8 vs 23.8, p = 0.03). Detailed results are summarized in Table 2.

We performed an additional analysis of changes in disease severity before and after bariatric surgery. OSA severity category decreased in 17 (38.6%) patients, increased in 2 (4.5%) patients and did not change in 18 (40.9%) patients. Normalization of sleep disturbances was observed in 7 (15.9% patients).

**Figure 4:**
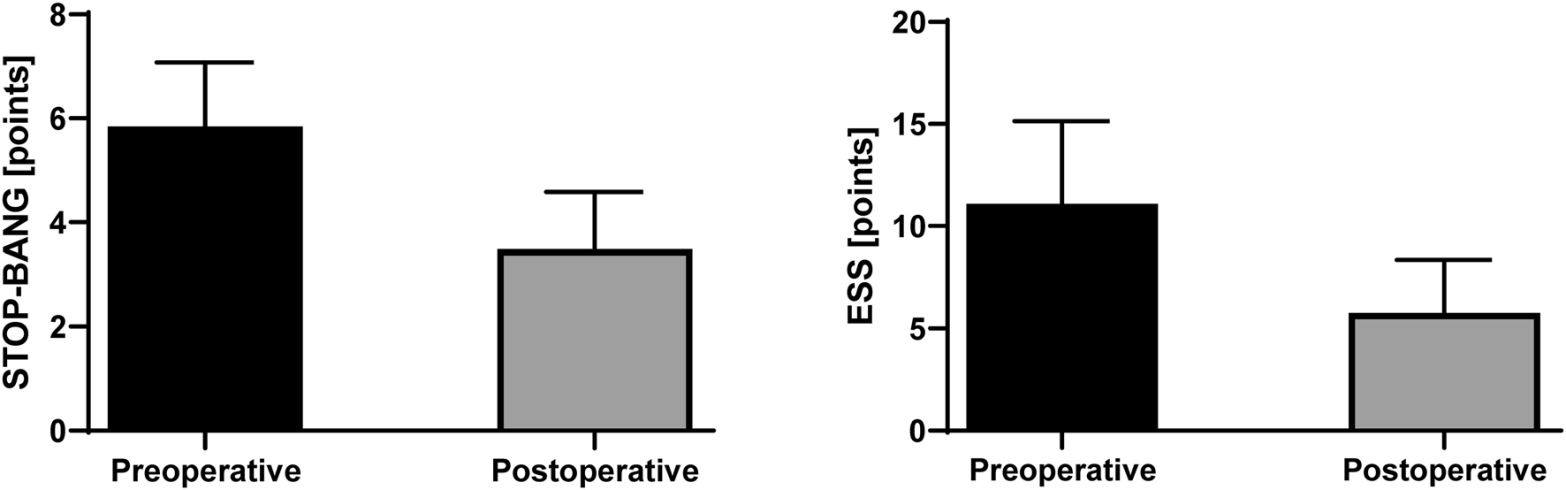
Perioperative changes in STOP-BANG and Epworth Sleepiness Scale scores. ESS, Epworth Sleepiness Scale.

We observed significant decrease in both STOP-BANG (6.0 vs. 3.0, p < 0.001) score and Epworth Sleepiness Scale (12.0 vs. 5.0, p < 0.001) as well as decrease in proportion of patients assessed as high risk according to Berlin questionnaire (93.2% vs. 29.5%). Excessive daytime sleepiness, defined as ≥ 10 points in ESS, was present in 30 patients preoperatively (68.2%) and in 3 patients after bariatric surgery (6.8%). Differences in scales scores before and after surgery are presented in Fig. 4.

### CPAP compliance and effectiveness

Compliance reports from patients’ CPAP devices were available in 36 patients. Median percentage of days with CPAP use accounted to 49.3% (15.8–76.0%), among which median percentage of days with CPAP use exceeding 4 h a day was 87.3% (35.9–97.3%). Effectiveness of therapy was reflected by median AHI during CPAP use accounting to 1.2 (0.4–2.3). We observed a significant reduction in therapeutic CPAP values (9.0 vs 6.3, p < 0.001) during re-titration with automatic CPAP coupled polysomnography.

## Discussion

In this prospective observational study, including 148 patients undergoing elective bariatric surgery, OSA was diagnosed in over 75% individuals, with the majority of cases being severe. Based on postoperative polysomnography, performed in 44 individuals, we demonstrated that bariatric surgery does not sufficiently reduce OSA, despite certain improvement in disease symptoms as perceived by patients, which possibly translates to premature discontinuation of CPAP therapy. In this study we showed that patients with OSA who underwent bariatric surgery require postoperative reassessment in order to evaluate indications for CPAP therapy, its effectiveness and patients` compliance.

Polysomnography performed one year after index surgery demonstrated that bariatric surgery significantly reduces respiratory disturbances during sleep but does not entirely eliminate OSA. Normalization of respiratory disturbances during sleep was observed in only 16% of patients while severity of OSA increased, remained stable, and decreased without normalization in 5%, 39%, and 41% respectively. In the presented study we attempted to assess factors potentially associated with the success of bariatric surgery in terms of decrease in AHI. Our results suggest that the proportion of excess AHI lost after the index surgery is moderately associated with postoperative reduction in excess BMI and weakly negatively correlated with age, however we found no significant relation with sex and predominance of hypopneas or apneas. Further studies should focus on search for factors predicting failure of bariatric surgery in improving sleep disturbances, because it may optimize the selection of patients requiring particular attention of sleep medicine specialists in the follow-up. Impact of bariatric surgery on OSA was evaluated in number of systematic reviews^9,20–23^ and original studies^24–29^, including randomized controlled trials^30,31^, concluding that majority of OSA patients do not achieve OSA resolution after bariatric surgery. The postoperative AHI shown in presented study is generally consistent with available literature, already mentioning the problem of residual OSA, however its extent seems to be higher than previously reported. This is probably related to higher baseline AHI values and higher prevalence of severe OSA in our cohort, compared to previously published papers^9,20–29^.

One of the main advantages of our study is comprehensive evaluation of perioperative changes in all polysomnography parameters, while the majority of available data is based solely on perioperative changes in AHI, patient-reported OSA remission or sleep questionnaires results. There is limited data documenting the influence of bariatric surgery on sleep architecture, since vast majority of authors analysed only respiratory PSG parameters without appropriate evaluation of perioperative changes in sleep architecture. In the present study we found that postoperative weight loss improves sleep quality, which is reflected by a significant improvement of sleep efficiency and decrease in number of respiratory related arousals and total number of arousals.

In parallel to improvement in sleep architecture, we found that bariatric surgery was associated with marked alleviation of excessive daytime sleepiness (EDS) quantified with Epworth Sleepiness Scale, reduction of OSA symptoms based on STOP-BANG questionnaire as well as decrease in proportion of patients assessed as high risk according to Berlin questionnaire. It is estimated that EDS is present in about 30% of obese individuals with BMI > 35 kg/m^2^ and might be independent from OSA presence^32^. The postulated EDS and obesity relationship could be explained by multiple pathophysiological mechanisms including overactivity of autonomic nervous system, hormonal and metabolic changes and obesity-related medical conditions^33–35^. Impact of weight loss on aforementioned pathways could partially clarify beneficial impact of bariatric surgery on EDS. Interestingly, there is data suggesting that the phenomenon of dramatic improvement of EDS related to weight reduction may occur regardless of OSA resolution^34,35^. The results of presented study might support the theory that EDS resolution does not fully correspond to change in apnea severity, since we demonstrated that bariatric surgery actually reduces EDS without satisfactory resolution of respiratory disturbances. Thus, diminished EDS related to bariatric surgery might rather be a result of more efficient sleep with less arousals. It has also been suggested that EDS in obese patients may depend on the level of nocturnal hypoxia^32^. In our study we demonstrated that bariatric surgery was associated with significant improvement of median and minimal SpO_2_ values and total time with SpO_2_ value < 90%. Moreover, the observed reduction of hypoxia duration and number of desaturation events was much more prominent than perioperative AHI decrease. These findings raise the question about the validity of OSA classification based only on AHI, thus omitting severity of hypoxia, which undoubtedly plays crucial role in pathogenesis of OSA symptoms and comorbidity. In our opinion, presented results suggest the need for routine postoperative PSG in order to precisely re-evaluate the severity of respiratory disturbances at least among patients diagnosed with severe OSA regardless of the postoperative weight loss or patient-reported alleviation of symptoms.

The present study also addressed the issue of premature postoperative CPAP cessation and poor CPAP compliance in patients with OSA undergoing bariatric procedures. We confirmed the effectiveness of CPAP therapy in this population with significant improvement of all crucial PSG parameters. Patients diagnosed with OSA were recommended to adhere to CPAP treatment preoperatively with indication to continue therapy for at least 12 months after bariatric surgery. The compliance reports obtained from patients’ CPAP devices at follow up visit were available in 36 cases. We proved high effectiveness of CPAP therapy reflected by median AHI accounting to 1.2/h (0.4–2.3/h) during CPAP. Unfortunately, compliance defined by median percentage of days with CPAP use accounted to less than 50%. We believe that actual CPAP compliance was even lower since great number of patients were excluded from the final analysis due to lack of postoperative PSG. There are published data indicating poor long-term CPAP compliance and problem of premature treatment discontinuation in general OSA population^36,37^. This also applies to OSA patients treated with bariatric surgery. Moreover, patients with sleep apnea who underwent bariatric surgery tend to discontinue CPAP therapy more frequently than other OSA patients and this phenomenon must not be underestimated^38^. Some authors suggested that CPAP therapy discontinuation after bariatric surgery may have a negative impact on the obesity-related comorbidities and increase the risk of OSA-associated complications. The factor potentially influencing CPAP compliance may be a reduction of therapeutic CPAP values after bariatric surgery confirmed in postoperative CPAP titration performed simultaneously with PSG. Preoperatively titrated CPAP pressure may turn out to be too high after significant weight loss, leading to poor tolerance of CPAP therapy and its premature discontinuation. However, in the present study vast majority of patients were treated with automatic CPAP devices (APAP), thus eliminating the need to modify the therapeutic CPAP pressure during 12-month follow-up period in contrast to traditional fixed pressure CPAP devices. Despite this fact, the CPAP compliance remained poor. Thus, the question which PAP therapy is the most suitable for OSA patients undergoing bariatric surgery remains unanswered, due to lack of studies directly comparing compliance with different CPAP devices in this setting. That also clearly indicates that problem of CPAP cessation after BS is multifactorial and warrants further investigation.

The undoubted advantage of this study is detailed evaluation of study cohort in tertiary centre sleep laboratory including fully supervised polysomnography (PSG), availability of comorbidity history, results of several types of sleep questionnaires, and CPAP therapy compliance data. However, there is one important limitation. It must be emphasized that from initially enrolled 148 individuals with preoperative PSG only 44 (40%) attended 12-month follow up hospitalization with postoperative PSG, which is an interesting observation itself. It might indicate a necessity for more frequent and probably more effectively enforced follow-up visits after bariatric surgery accompanied by CPAP compliance analysis and identification of possible problems influencing CPAP therapy (value of pressure, mask fit, psychological factors affecting CPAP usage). Simultaneously we should pay careful attention to patient re-education emphasizing the risk of OSA complications potentially associated with premature CPAP cessation and fact of high frequency of persistent asymptomatic OSA.

## Conclusions

This study suggests that bariatric surgery does not sufficiently reduce obstructive sleep apnea what warrants mandatory postoperative polysomnography in order to precisely re-evaluate at least all severe OSA patients regardless of the postoperative weight loss or patient-reported resolution of symptoms. Moreover, sleep questionnaires are probably not a reliable tool for evaluation of persistent OSA after bariatric surgery. Patients with OSA undergoing bariatric surgery are characterized by poor CPAP compliance what indicates the need for proper education and early follow-up visits with identification of potential factors influencing the therapy.

## Data Availability

Data will be made available upon request.

## Code availability

Statistical analysis was performed using Statistica 13 software.
